# Are perceptions of community safety associated with respiratory illness among a low-income, minority adult population?

**DOI:** 10.1186/s12889-018-5933-4

**Published:** 2018-09-03

**Authors:** Kristen N. Arthur, Rhonda Spencer-Hwang, Synnøve F. Knutsen, David Shavlik, Samuel Soret, Susanne Montgomery

**Affiliations:** 1 School of Public Health, Center for Community Resilience, Loma Linda University, 24951 N. Circle Drive, Nichol Hall, room 1401, Loma Linda, CA 92350 USA; 2 School of Public Health, Center for Nutrition, Health Lifestyle and Disease Prevention, Loma Linda University, Loma Linda, USA; 3 School of Behavioral Health, Loma Linda University, Loma Linda, USA

**Keywords:** Community health, Asthma, Chronic obstructive pulmonary disease (COPD), Violence, Perceptions, Community safety

## Abstract

**Background:**

Growing evidence suggests social disadvantage magnifies the harmful health effects of environmental hazards; however, there is limited research related to perceptions of risk among individuals who live near such environmental hazard sites. We explored the association between individual-level perception of community safety and respiratory illness among low-income, minority adults who live in a region with routine poor air quality exacerbated by the emissions of a nearby freight railyard.

**Methods:**

Interview-administered household surveys were collected (87% response rate; *n* = 965) in English/Spanish from varying distances surrounding a freight railyard (analytic total *n* = 792: nearest region *n* = 215, middle *n* = 289, farthest *n* = 288). Illness outcome was an affirmative response to doctor-diagnosed asthma, bronchial condition, emphysema, COPD, or prescribed-inhaler usage. Respiratory symptoms outcome was an affirmative response to chronic cough, chronic mucus, or wheezing. The independent variable was perceived community safety.

**Results:**

Outcome prevalences were similar across environmental hazard regions; 205 (25.9%) were diagnosed-illness cases and 166 (21.0%) diagnosis-free participants reported symptoms. Nearly half (47.5%) of participants reported lack of perceived community safety, which was associated with environmental hazard region (*p* <  0.0001). In multivariable log-binomial regression models adjusting for covariables (age, gender, race/ethnicity, smoking status, smoke exposure, residential duration, and distance from the railyard) respiratory illness diagnosis was associated with lack of perceived community safety (PR = 1.39; 95% CI 1.09, 1.76). Sensitivity analyses showed a non-significant but increasing trend in the strength of association between safety perceptions and illness diagnoses with closer proximity to the railyard.

**Conclusions:**

Our findings contribute to the literature that individuals’ perceptions of community safety are associated with adverse respiratory health among a population living in high air pollution exposure areas.

**Electronic supplementary material:**

The online version of this article (10.1186/s12889-018-5933-4) contains supplementary material, which is available to authorized users.

## Background

Exposure to social stressors and environmental hazards are more common and are elevated in low-income, minority, urban communities [1–5]. While it is well established that higher polluting industries are more likely to settle near low income communities (or that low income communities develop nearby due to lower housing costs) [6, 7], there is also growing evidence that suggests that social disadvantage (e.g. exposure to community violence) magnifies the effects from environmental hazards on adverse health outcomes [8–15].

Stress appears to not only have a direct effect on health outcomes, but may also influence susceptibility to or be influenced by other determinants of health [16]. Animal studies support the biologic plausibility that chronic social stress leads to greater susceptibility of respiratory health issues due to air pollution exposure [17]. Because the effects of air pollution on respiratory health, especially asthma, have been found to be greater in low socio-economic status (SES) populations, psychosocial stress has been suggested to also have an effect on this relationship [12, 18]; this suggests that those who are exposed to both air pollution and social stress are more susceptible to the adverse health effects of air pollution than those who are singly exposed [11, 19].

Residential community violence/crime or an overall feeling of living in an unsafe neighborhood are measurable factors of social disadvantage. Neighborhood-level violence/crime, as a measure for psychosocial stress, is a suggested risk factor in the pathophysiology of asthma incidence and morbidity [20, 21]. Perceived stress may also be a risk factor for adult-onset asthma [22] and has been strongly associated with increased asthma morbidity [23]. Exposure to violence, measured individually, has been consistently associated with worsening asthma morbidity in children [10, 24, 25], risk of asthma among children [11, 26], and asthma symptom severity in adults [27, 28]. In addition, the Adverse Childhood Experiences Study has found a graded and significant relationship between exposure to violence/trauma during childhood and incidence and morbidity of chronic obstructive pulmonary disease (COPD) as an adult [29].

Within the context of environmental hazards research, the effects on adverse respiratory health from air pollution may have become diluted in analyses when whole communities with healthy individuals are investigated. For instance, a greater and more pertinent effect on adverse health may be found when instead investigating vulnerable populations from both perspectives - either as environmentally vulnerable (e.g. those living very near high traffic roads or goods movement traffic) or as socially vulnerable (e.g. those who are more exposed to psychosocial stressors) [30, 31]. Research on the effects of social disadvantage and ambient air pollution on respiratory health is specifically lacking in adult populations living in the context of this type of intersection (social and environmental vulnerability), as is the case for minority populations living near a major pollutant emission source, such as a freight railyard.

The inland region of Southern California offers such a yet to be studied context. In a region well known for routine poor air quality [32], it is also home to the San Bernardino Railyard (SBR), a major stationary, diesel particulate emitting matter source [7], located adjacent to a densely populated low income community. This SBR region is on a shortlist of widely recognized full-fledged inland ports in the U.S. with diesel-powered locomotives and trucks operating 24/7 [7, 33]. According to the California Air Resources Board Health Risk Assessment report, the diesel particulate matter emissions within one mile from the SBR are estimated at about 22 tons per year, which represent 66% of the total on-site and off-site emissions combined [33]. Of 18 railyards in California, the SBR has the highest population exposure to railyard emissions due to the highest residential density near the railyard [7]. Moreover, San Bernardino County residents continuously exhibit higher chronic disease morbidity than California state counterparts, including asthma [34]. San Bernardino County is one of the most underfunded regions in the state [35]. It’s economic hardship became well known after the city filed for bankruptcy in 2012 [36]. Nearer to the SBR, the city of San Bernardino has the third largest number of gang members in the U.S. [35]. In 2012, metropolitan San Bernardino had more than twice the violent crime and murder offenses per population size compared to neighboring Los Angeles [37] and the city in past decades has consistently been in the top 25 most violent cities in the U.S. [38]. Many other social determinants from economic to neighborhood segregation support evidence that this is a socially and environmentally vulnerable population exhibiting varying levels of social deprivation [35].

Households from the San Bernardino city area directly adjacent to, and at varying distances from, this goods movement network were included in the Environmental Railyard Research Impacting Community Health (ENRRICH) Project, a mixed-methods, community-based participatory research study. During the community engagement phase of the research project, community members expressed that most of their immediate concerns centered on issues related to law enforcement, street lighting and repair, trees and greenery, and a violence and unemployment ripple effect [39]; in other words, social and neighborhood health factors, as opposed to personal health concerns such as asthma due to the nearby emitting railyard. This paper explores follow-up survey results of adult residents. The goal was to explore if the social stressor related concerns community members had (i.e. perceived community safety) were related to adverse respiratory health outcomes. Thus, in a predominantly low-income, minority, environmentally vulnerable adult population, this paper aims to determine (1) the extent to which this population perceives their community as unsafe and (2) to what degree such perceptions affect respiratory illness.

## Methods

### Study population

The ENRRICH Project was conducted in 2011–2012 by Loma Linda University (LLU) researchers and a local community partner organization (Center for Community Action and Environmental Justice), using community-based participatory methods to reach the hard-to-access, low income, predominantly Latino population living near the San Bernardino Railyard (SBR). The purpose was to characterize the community health burden of disease in the residential areas near the SBR, an inland goods movement network with routine, severe ambient air pollution problems. To account for the seasonal variation in local air quality, ENRRICH investigators conducted two cross-sectional waves of data collection, one in the summer of 2011 and a second wave in the winter-spring of 2012.

**Fig. 1:**
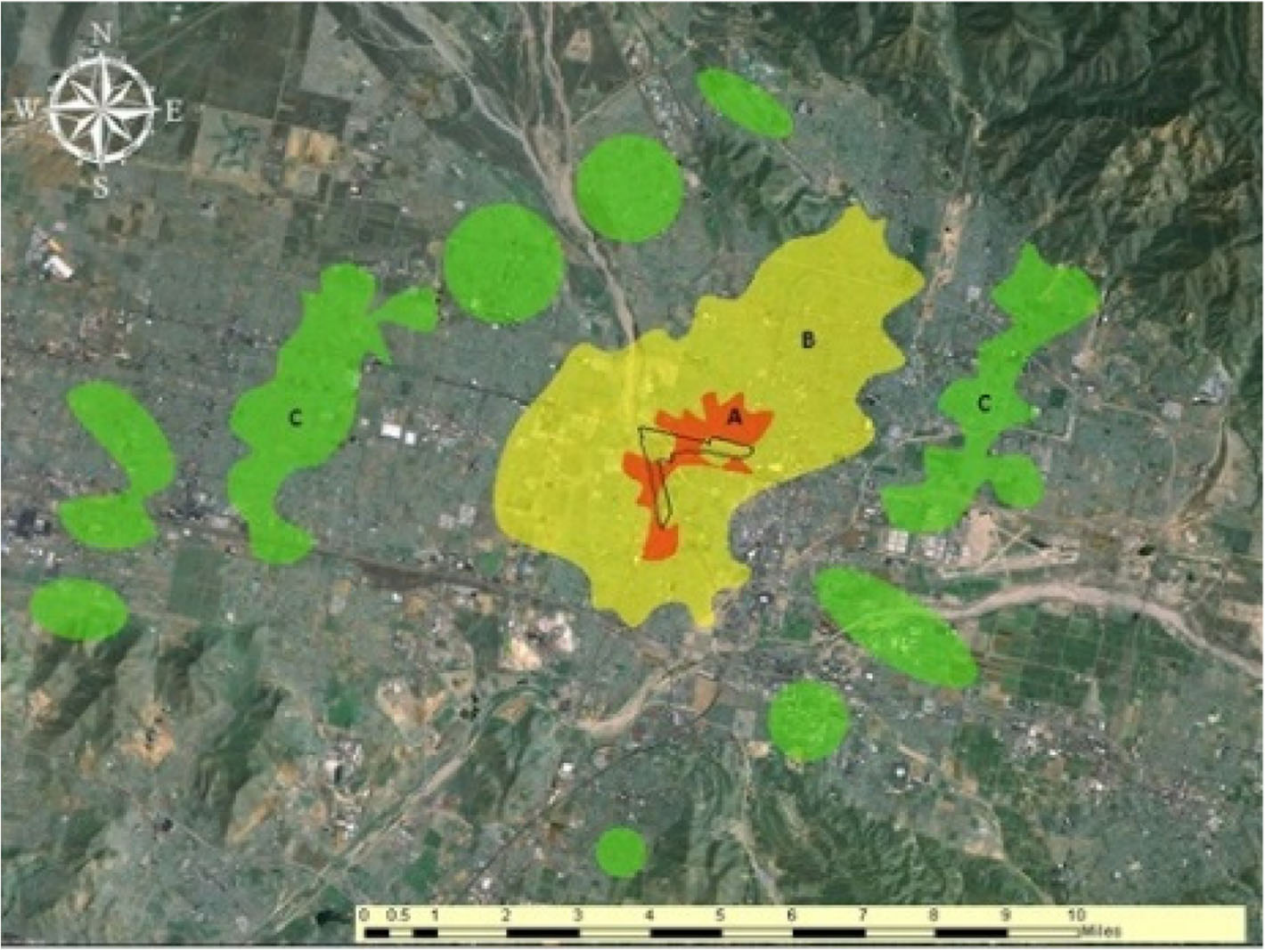
Geography of the BNSF San Bernardino Railyard in southwestern San Bernardino County, inland Southern California. Railyard outlined in black. Sampling regions (also referred to as, environmental hazard regions) from which households were selected: A (nearest region, red); B (middle region, yellow); and C (farthest region, green)

Three sampling regions (A, B, and C) surrounding the SBR were surveyed using a community-based participatory research (CBPR) approach; a CBPR approach incorporates (in our case) carefully trained community members alongside research investigators (who are often academic) during development, administration, and analysis of a study/project. The survey data were collected from adults (ages 18 years and older) present in the household at the time of survey. The location and spatial configuration of the sampling regions are depicted in Fig. 1. The three regions were designed to model decreasing levels of air pollution exposure (derived through computer-based air dispersion modeling based on the California Air and Resource Board’s Health Risk Assessment) in relation to the railyard, from highest (A) to lowest (C). We will refer to these three sampling or residential regions as environmental hazard regions.

Region A was defined by delineating a 350-m buffer around the perimeter of the railyard facility and every house within region A was considered for interviewing. Within sampling regions B and C, households were randomly selected - ENRRICH investigators used digital street and cadastral maps of the target neighborhoods and assigned households for interviews using a GIS-based random number generator tool. Sampling methodology with regards to air dispersion modelling is discussed more in depth elsewhere [35]. For safety reasons, interviewing was conducted only during daylight hours; this included early evenings and weekends. There were 965 complete interviews (300 in region A, 338 in region B, 324 in region C) and 792 had complete information for all analyzed variables thus constituting our analytic study population (215 in region A, 289 in region B, 288 in region C).

### Survey

LLU investigators utilized a CBPR approach to the study design with a mixed methods research methodology to develop the survey instrument. To gather information in a culturally competent and linguistically-appropriate manner, the survey instrument was developed based on mixed methods research exploring the perceived needs and challenges of the affected population, as well as from existing literature. The process included internal technical discussions with the community-based partners and direct community feedback in the form of in depth key informant interviews and focus groups. This mixed-methods process helped to identify relevant questions (from both scientific and community perspectives) to incorporate into the survey, such as lived experiences as well as attitudes and perceptions regarding the health impacts from residential proximity to the SBR and is reported elsewhere [39].

Once developed, the survey was translated to Spanish and pilot tested. It consists of several sections including questions pertaining to demographics, description of household socioeconomic status, history of doctor-diagnosed illnesses, respiratory symptoms, hearing impairment, use of medications, health care utilization, occupational and residential histories, smoking status/history, indoor sources of air pollution, and perceptions of their community (Additional file 1). Survey administration, in a personal interview format to address low reading levels, was delivered by trained, bi-lingual community health workers (as part of our CBPR approach) from the study’s local community partner, a non-profit environmental justice organization [40].

The research study was approved by Loma Linda University Institutional Review Board (IRB #5110054). All participants provided informed written consent prior to participation in the study.

### Perceived community safety stressor

The perceived community safety independent variable used in this paper is a construct created from two survey items pertaining to community safety on the ENRRICH household survey: “*I feel safe walking in my community, day or night.*” and “*Violence or crime is not a problem in my community.*” Participants responded on a 5-point Likert scale from strongly agree to strongly disagree. For ease of interpretation, responses to each of the two statements were dichotomized. A response of “disagree” or “strongly disagree” was considered as the exposure (i.e. lack of perceived community safety as the exposure). We then created a dichotomous perceived community safety construct variable to capture any perception of lack of community safety. The exposed category was defined as responding “disagree” or “strongly disagree” to either one of the above two statements.

### Respiratory illness outcomes

Two respiratory health endpoints were assessed in this study: self-reported doctor-diagnosed respiratory illness and self-reported chronic respiratory symptoms. Both outcome variables were created constructs from the ENRRICH household survey.

The self-reported doctor-diagnosed respiratory illness outcome was created from survey questions that asked if the participant ever had a doctor-diagnosis of asthma, a bronchial condition, emphysema, or chronic obstructive pulmonary disease (COPD). Pilot testing indicated that some respondents had low health care literacy and did not clearly understand the term “diagnosis.” For this reason, use of an inhaler (“*Do you use a physician-prescribed inhaler?*”) was asked to determine if there were participants who were prescribed medication for respiratory conditions, but said “no” to ever being diagnosed with a chronic respiratory illness. Participants were counted as a respiratory illness case if they had replied “yes” to any one of the respiratory illnesses or to use of a prescribed inhaler.

Because some participants may not have been diagnosed yet with a respiratory illness due to low-income status and/or limited health care access, we also included self-reported respiratory symptoms as an outcome for analysis among the subset of the participants who did not report a respiratory illness. The self-reported chronic respiratory symptoms outcome includes current chronic coughing and current chronic mucus (defined as having the symptom “*on most days for three months or more out of the year*”), and ever having wheezing not due to a cold. Participants responded a dichotomous yes/no to these questions. Participants were counted as a respiratory symptoms case if they had replied “yes” to any one of the three symptoms.

### Statistical analyses

For descriptive statistics, we computed frequencies for categorical variables and mean and standard deviation for continuous variables (i.e. age). For bivariate associations, we computed chi-square tests for categorical variables and a two-sample t-test for the variable age, which was normally distributed.

We studied the association between perceived lack of community safety stressors and the outcomes (respiratory illness and respiratory symptoms constructs) using multivariable log-binomial regression models which allowed for the calculation of prevalence ratios (PR) and 95% confidence intervals (95% CI) [41]. Covariates were a priori selected based on relevance in the literature and availability within the ENRRICH dataset. The minimally-adjusted model included risk factors of the respiratory disease outcome [42–45]: age, gender (male, female), race/ethnicity (Hispanic/Latino; black/African-American; white, Asian, other), smoking status (currently, past, never), having lived with a smoker for more than six months as an adult (currently, past, never), duration at current residence (< 1 year, 1–10 years, ≥ 11 years), and environmental hazard exposure region (A, B, C). In addition to the minimally-adjusted model variables, the final model added the following individual-level socio-economic status variables: health care insurance (none; private, blue cross, HMO, military or other; Medicare, Medicaid, or Medi-cal), employment status (unemployed; part-time employed, full-time employed, retired or student), and number of people living at the current residence (1–2 people, 3–5 people, ≥ 6 people).

We chose not to collapse the covariate duration at current residence for less than one year, despite a small count, to reflect a possible biological effect, but interpreted statistics for duration at current residence with caution. Education was not included as a covariate because of its high correlation with unemployment, insurance, and number in household; in addition, adding education to the model did not change the precision of model and did not statistically confound the relationship between the respiratory illness outcome and community safety variable. We considered income as a potential confounder but chose to not include it in the full multi-variable models due to its high number of missing (*n* = 93 missing). To assess its affect in the full multi-variable model, we conducted a sensitivity analysis with income in the full model and found that the main effect between respiratory illness and perceived community safety did not change.

To further describe the association between perceived community safety and respiratory illness, we conducted sensitivity and effect modification analyses by region. Sensitivity analyses included region specific analyses using the same multivariable models stated above. Results are interpreted with caution due to smaller sample sizes. Our effect modification analyses tested multiplicative interaction between the perceived community safety construct and environmental hazard region within the multivariable fully-adjusted model with respiratory illness construct as the outcome. Environmental hazard region was included into the models as a nominal (A, B, and C) and dichotomous (A/B and C) variable. The choice to collapse regions A and B was to keep the regions closer to the freight railyard together in comparison to region C, which is the region on the edge of the metropolitan San Bernardino city area.

In our last sensitivity analysis, we tested the association between perceived community safety and respiratory symptoms construct only among the subgroup of individuals who did not report a respiratory illness. SAS version 9.4 was used for all analyses.

## Results

### Study population characteristics

The ENRRICH study population was predominantly Hispanic (76%), female (68%), married (58%) and high school educated or less (61%). Participants reported having low income, 93% of the study population reported an average annual household income of less than $50,000 with 86% reporting a household size of three or more persons. Coinciding with a low self-reported average annual household income, 77% of participants reported having either no health insurance or being covered by Medicaid, Medicare, or Medi-cal. The majority of participants (94.6%) had lived at their current residence for more than one year (Table 1).

**Table 1:** Study population characteristics by respiratory illness status and by lack of perceived community safety construct

Variable		Respiratory Illness			Lack of Community Safety		
	Total	Yes	No		Yes	No	
	(*n* = 792)	(*n* = 205)	(*n* = 587)	*p*-value	(*n* = 376)	(*n* = 416)	*p*-value
	n (%)	n (%)	n (%)		n (%)	n (%)	
Age, mean ± SD	44.4 ± 14.3	46.3 ± 14.8	43.8 ± 14.0	0.03*	44.6 ± 14.1	44.3 ± 14.4	0.77
Gender							
Female	540 (68.2)	138 (67.3)	402 (68.5)	0.78	262 (69.7)	278 (66.8)	0.39
Male	252 (31.8)	67 (32.7)	185 (31.5)		114 (30.3)	138 (33.2)	
Employment status							
Unemployed	368 (46.5)	89 (43.4)	279 (47.5)	0.31	176 (46.8)	192 (46.2)	0.85
Employed, student or retired	424 (53.5)	116 (56.6)	308 (52.5)		200 (53.2)	224 (53.8)	
Race/Ethnicity							
Hispanic/Latino	606 (76.5)	140 (68.3)	466 (79.4)	< 0.01*	279 (74.2)	327 (78.6)	0.03*
African-American/Black	85 (10.7)	31 (15.1)	54 (9.2)		37 (9.8)	48 (11.5)	
White, Asian, Other	101 (12.8)	34 (16.6)	67 (11.4)		60 (16.0)	41 (9.9)	
Education							
≤ Grade school	225 (28.7)	66 (32.7)	159 (27.4)	0.50	113 (30.5)	112 (27.1)	0.26
High School	253 (32.3)	59 (29.2)	194 (33.4)		122 (33.0)	131 (31.7)	
Some college, vocational, business, or trade	232 (29.6)	58 (28.7)	174 (29.9)		108 (29.2)	124 (30.0)	
≥ Associates degree	73 (9.3)	19 (9.4)	54 (9.3)		27 (7.3)	46 (11.1)	
Health care insurance							
None	355 (44.8)	73 (35.6)	282 (48.0)	< 0.01*	178 (47.3)	177 (42.5)	0.29
Private, Blue Cross, HMO, military, or other	183 (23.1)	51 (24.9)	132 (22.5)		87 (23.1)	96 (23.1)	
Medicare/caid/cal	254 (32.1)	81 (39.5)	173 (29.5)		111 (29.5)	143 (34.4)	
Ever regularly smoked cigarettes, cigars, or a pipe							
Current smoker	153 (19.3)	54 (26.3)	99 (16.9)	0.01*	79 (21.0)	74 (17.8)	0.51
Past smoker	92 (11.6)	20 (9.8)	72 (12.3)		42 (11.2)	50 (12.0)	
Never smoker	547 (69.1)	131 (63.9)	416 (70.9)		255 (67.8)	292 (70.2)	
As an adult, lived for > 6 mo. with someone who smoked							
Yes, currently	31 (3.9)	12 (5.9)	19 (3.2)	0.07	16 (4.3)	15 (3.6)	0.45
Yes, in the past	184 (23.2)	55 (26.8)	129 (22.0)		94 (25.0)	90 (21.6)	
No	577 (72.9)	138 (67.3)	439 (74.8)		266 (707)	311 (74.8)	
Average annual household income							
< $10,000	214 (30.6)	55 (30.9)	159 (30.5)	0.82	111 (32.4)	103 (28.9)	0.11
$11,000 - $19,000	175 (25.0)	49 (27.5)	126 (24.2)		96 (28.0)	79 (22.2)	
$20,000 - $29,000	138 (19.7)	31 (17.4)	107 (20.5)		65 (19.0)	73 (20.5)	
$30,000 - $49,000	125 (17.9)	30 (16.9)	95 (18.2)		53 (15.4)	72 (20.2)	
> $50,000	47 (6.7)	13 (7.3)	34 (6.5)		18 (5.2)	29 (8.2)	
Marital Status							
Never married/ widowed/divorced	328 (41.5)	106 (51.7)	222 (38.0)	< 0.001*	166 (44.4)	162 (38.9)	0.12
Married/live together	462 (58.5)	99 (48.3)	363 (62.0)		208 (55.6)	254 (61.1)	
People in household							
1–2 people	109 (13.8)	41 (20.0)	68 (11.6)	< 0.001*	59 (15.7)	50 (12.1)	0.30
3–5 people	430 (54.3)	116 (56.6)	314 (53.5)		202 (53.7)	228 (54.8)	
6 or more people	253 (31.9)	48 (23.4)	205 (34.9)		115 (30.6)	138 (33.1)	
Duration at current residence							
11+ years	213 (26.9)	57 (27.8)	156 (26.6)	0.09	105 (27.9)	108 (26.0)	0.14
1–10 years	536 (67.7)	143 (69.8)	393 (66.9)		245 (65.2)	291 (69.9)	
< 1 year	43 (5.4)	5 (2.4)	38 (6.5)		26 (6.9)	17 (4.1)	
Environmental hazard region							
A - closest to SBR	215 (27.1)	50 (24.4)	165 (28.1)	0.35	131 (34.8)	84 (20.2)	< 0.0001*
B - intermediate	289 (36.5)	83 (40.5)	206 (35.1)		136 (36.2)	153 (36.8)	
C - farthest away	288 (36.4)	72 (35.1)	216 (36.8)		109 (29.0)	179 (43.0)	

*Denotes a statistically significant *p*-value for chi-square statistic (or t-test for the variable age) at alpha-level ≤ 0.05

% = column percent; SD = standard deviation; n (%) for categorical variables and mean ± SD for continuous variables (i.e. age); SBR = San Bernardino Railyard

### Perceived community safety

Two-hundred twenty-five (28.4%) participants reported feeling unsafe walking in their community, 309 (39.0%) reported violence/crime to be a problem in their community, and 376 (47.5%) reported a lack of perceived community safety as measured by the construct variable. Of those who reported lack of perceived community safety, 42.0% (*n* = 158) reported “yes” to both feeling unsafe walking and violence/crime to be a problem in their community.

Those who lacked perceived community safety, as measured by the construct, were more likely to self-report being White, Asian or “other” (*p* = 0.03) and to live nearest to the environmental hazard (*p* <  0.0001) (Table 1).

### Respiratory illness outcome

Two-hundred five participants (25.9%) were considered as a doctor-diagnosed respiratory illness case as measured by the constructed respiratory illness variable which included inhaler-use. One-hundred ninety (24.0%) participants reported respiratory illness (asthma, emphysema, chronic bronchitis or COPD). Asthma was the most reported respiratory illness (*n* = 98) followed by a bronchial condition (*n* = 93), COPD (*n* = 34), and emphysema (*n* = 8). Twenty-seven participants (3.4%) reported at least two different respiratory illnesses and ninety (11.3%) participants reported use of a physician-prescribed inhaler. (Table 2). Lastly, of those who reported any respiratory illness, 39.4% (*n* = 74) also reported use of a prescribed inhaler, the majority of whom reported doctor diagnosis of asthma (*n* = 58).

**Table 2:** ENRRICH Study population respiratory illness prevalence by perceived lack of community safety construct

	Total	Lack of perceived community safety construct		
		Yes	No	*p*-value
Respiratory illness variables Among total study population				
	*n* = 792 n (%)	*n* = 376 n (%)	*n* = 416 n (%)	
Respiratory Illness Construct^a^				
Yes	205 (25.9)	115 (30.6)	90 (21.6)	< 0.01*
No	587 (74.1)	261 (69.4)	326 (78.4)	
Doctor-diagnosed asthma, bronchial condition, emphysema, or COPD				
Yes	190 (24.0)	108 (28.7)	82 (19.7)	< 0.01*
No	602 (76.0)	268 (71.3)	334 (80.3)	
Ever doctor-diagnosed respiratory illnesses (not mutually exclusive):				
Asthma	98 (12.4)	52 (13.8)	46 (11.1)	0.24
Bronchial condition	93 (11.7)	56 (14.9)	37 (8.9)	< 0.01*
Emphysema	8 (1.0)	6 (1.6)	2 (0.5)	0.12
COPD	34 (4.3)	21 (5.6)	13 (3.1)	0.09
Two or more of the above	27 (3.4)	17 (4.5)	10 (2.4)	0.10
Medication Use				
Prescribed-inhaler use	90 (11.3)	55 (14.7)	35 (8.4)	< 0.01*
Respiratory Symptoms Variables Among Respiratory Illness Free Sub-Population				
	*n* = 587 n (%)	*n* = 261 n (%)	*n* = 326 n (%)	
Respiratory Symptoms Construct^b^				
Yes	166 (29.0)	79 (31.1)	87 (27.3)	0.32
No	407 (71.0)	175 (68.9)	232 (72.7)	
Chronic Cough				
Yes	91 (15.6)	47 (18.1)	44 (13.5)	0.13
No	494 (84.4)	213 (81.9)	281 (86.5)	
Chronic Mucus				
Yes	85 (14.6)	43 (16.5)	42 (13.1)	0.24
No	496 (85.4)	217 (83.5)	279 (86.9)	
Ever had wheezing				
Yes	77 (13.4)	40 (15.8)	37 (11.6)	0.15
No	496 (86.6)	214 (84.2)	282 (88.4)	
Two or more of the above				
Yes	65 (11.1)	37 (14.2)	28 (8.6)	0.03*
No	522 (88.9)	224 (85.8)	298 (91.4)	

*Denotes a statistically significant *p*-value at alpha-level ≤ 0.05 from chi-square statistic

^a^Construct created from survey questions asking if participant had doctor-diagnosed respiratory illness (asthma, bronchial condition, emphysema, or COPD) or used a prescribed-inhaler

^b^Construct created from survey questions asking if participant had chronic respiratory symptoms (chronic cough, chronic mucus, or ever had wheezing not due to a common cold)

The proportion of self-reported doctor diagnosed respiratory illness outcome did not differ by environmental hazard (residential) region. Those with self-reported doctor diagnosed respiratory illness outcome were more likely to be non-Hispanic (*p* = 0.005), have some form of health insurance (*p* = 0.006), be a current smoker (*p* = 0.01), single (never married/widowed/divorced) (*p* = 0.0006), or live alone or with one other person (*p* = 0.0008) (Table 1).

Doctor-diagnosis of a respiratory illness as measured by the construct (*p* = 0.004), any doctor-diagnosed respiratory illness (asthma, bronchial condition, emphysema, or COPD) (*p* = 0.003), a doctor-diagnosis of bronchial condition (*p* = 0.009) and prescribed-inhaler use (p = 0.006) were each associated with lack of perceived community safety (Table 2).

#### Log-binomial regression analyses

The prevalence of participants with a self-reported doctor-diagnosed respiratory illness was 40% greater among the group of participants who reported lack of perceived community safety (PR = 1.39; 95% CI 1.09, 1.76). The strengths of the associations were similar for both stressor variables, feeling unsafe walking (PR = 1.37; 95% CI 1.07, 1.74) or perceiving violence/crime to be a problem in their community (PR = 1.36; 95% CI 1.07, 1.72), compared to the community safety construct variable (Table 3). In the fully-adjusted model with community safety construct as the independent variable (Table 3), duration of residence for less than one year and reporting Medicare, Medicaid, or Medi-cal health insurance were also associated with a higher prevalence of self-reported respiratory illness (PR = 0.38, 95% CI 0.17, 0.87 versus 1–10 years; PR = 1.45, 95% CI 1.10, 1.91 versus no health insurance).

**Table 3:** Log-binomial regression modeling of association between perceived community safety stressors and self-reported doctor-diagnosed respiratory illness

Independent variable	Crude model		Minimally-adjusted model		Fully-adjusted model	
	PR (95% CI)	*p*-value	PR (95% CI)	*p*-value	PR (95% CI)	*p*-value
Total Population						
“I do not feel safe walking in my community, day or night.”	1.31 (1.02, 1.67)	0.03*	1.34 (1.05, 1.71)	0.02*	1.37 (1.07, 1.74)	0.01*
“Violence or crime is a problem in my community.”	1.38 (1.09, 1.74)	< 0.01*	1.34 (1.06, 1.70)	0.02*	1.36 (1.07, 1.72)	0.01*
Lack of perceived community safety construct	1.41 (1.11, 1.79)	< 0.01*	1.39 (1.10, 1.77)	< 0.01*	1.39 (1.09, 1.76)	< 0.01*
Region A Only						
Lack of perceived community safety construct	1.83 (1.03, 3.23)	0.04*	1.98 (1.09, 3.58)	0.02*	1.79 (0.97, 3.28)	0.06
Region B Only						
Lack of perceived community safety construct	1.47 (1.02, 2.12)	0.04*	1.44 (1.00, 2.07)	0.05	1.41 (0.98, 2.04)	0.06
Region C Only						
Lack of perceived community safety construct	1.24 (0.83, 1.85)	0.29	1.12 (0.75, 1.69)	0.58	1.14 (0.75, 1.72)	0.55
Regions A & B						
“I do not feel safe walking in my community, day or night.”	1.48 (1.10, 1.98)	< 0.01*	1.54 (1.15, 2.06)	< 0.01*	1.59 (1.18, 2.14)	< 0.01*
“Violence or crime is a problem in my community.”	1.43 (1.07, 1.91)	0.02*	1.44 (1.07, 1.93)	0.02*	1.43 (1.07, 1.93)	0.02*
Lack of perceived community safety construct	1.52 (1.12, 2.07)	< 0.01*	1.56 (1.15, 2.11)	< 0.01*	1.52 (1.12, 2.07)	< 0.01*

*Denotes a statistically significant *p*-value at alpha-level ≤ 0.05 from chi-square statistic

Referent category for each independent variable is not shown in the Table (PR = 1.00)

Minimally-adjusted model covariables: age, gender, race/ethnicity, smoking status, smoke exposure, residential duration, environmental hazard region

Fully-adjusted model covariables: minimally-adjusted model + insurance status, unemployment, number of household members

PR = prevalence ratio, 95% CI = 95% confidence interval

We found an increasing, but non-significant, trend in the strength of associations between lack of perceived community safety and respiratory illness as residents lived closer to the freight railyard (Table 3). For subpopulation regions A & B, the association between lack of perceived community safety and respiratory illness persisted (PR = 1.52, 95% CI 1.12, 2.07), although the confidence interval also overlaps with that of the total population (Table 3). The multiplicative interaction term between the perceived community safety construct and environmental hazard region was not significant in the fully-adjusted model for the total population.

### Respiratory symptoms outcome

Among the subgroup of participants who did not report a respiratory illness (*n* = 587), 166 participants (29.0% or 21.0% of the total population) reported respiratory symptoms measured as a construct. Ninety-one (15.6%) reported chronic cough, 85 (14.6%) reported chronic mucus, and 77 (13.4%) reported wheezing symptoms not due to a cold. Of those who reported a respiratory symptom, 11.1% (*n* = 65) reported two or more symptoms (Table 2).

The proportion of self-reported respiratory symptoms outcome did not differ by environmental hazard (residential) region. Those who reported respiratory symptoms were more likely to have Medicare, Medicaid, or Medi-cal insurance (*p* = 0.016), be a current smoker (*p* = 0.014), and have lived with a smoker in the past (*p* = 0.002). Reporting two or more symptoms was associated with lack of perceived community safety (*p* = 0.032) (Table 2). In the fully-adjusted log-binomial model, lack of perceived community safety was not associated with the prevalence of respiratory symptoms (PR = 1.14, 95% CI 0.89, 1.46).

## Discussion

To our knowledge, this is the first study to provide insight into the possible effect of community perceptions of safety on respiratory illness among adult, low-income, minority community members who were also environmentally vulnerable due to living in a region with routine poor air quality and with the additional exposure to a major pollutant source. We found a strong independent association between lack of perceived community safety and doctor diagnosed respiratory illness. Perceiving your community to be unsafe, arguably a stressful feeling, increased the likelihood of having been diagnosed with a respiratory illness by 40%. This effect was independent from other known factors associated with respiratory illness, such as age, tobacco exposure, low socio-economic status, or even distance from a diesel emission particulate matter air polluter. Although no association was found between perceived community safety and respiratory symptoms among the un-diagnosed population, these 166 participants with symptoms but no respiratory illness (21% of the total population) may represent future diagnosed respiratory illness cases in this population. Our findings are in line with others who have found that psychosocial stressors, such as perceived community safety, may contribute to chronic stress which in turn may contribute to the development [29, 46] and/or exacerbations [23, 28] of chronic respiratory illnesses, including asthma and COPD. Chronic stress is known to affect the immune and pro-inflammatory systems, which are suspected to be a part of the etiology of asthma [17, 22, 47–49].

Studies of the effects of psychosocial stress, including exposure to violence [11, 24, 26, 28], on respiratory health have largely been conducted in children [10, 12, 13]. To our knowledge, this rigorously designed cross-sectional study of individually-measured, perceived lack of community safety on respiratory health among a socially- and environmentally- vulnerable adult population is the first of its kind. Our results are in line with a broader body of literature that supports evidence for the association between past exposure to trauma or violence/adversity, often measured by adverse childhood experiences, and chronic respiratory illness among adults [29, 46, 50–52]; however, these did not adjust for ambient air pollution indicators as was did by adjusting for residential proximity to the railyard.

In addition, in line with our previously published qualitative findings [39] we found that a perception that one’s residential community is unsafe was highly prevalent. More than one in four participants reported an unsafe walking environment and more than one in three participants perceived a problem of community violence/crime demonstrating this population to be socially vulnerable. Perception of community safety was, not surprisingly, place-based; the closer the residential region was to the environmental hazard the higher the proportion of participants who reported lack of perceived community safety. Others have reported evidence supporting the causal relationship between upstream, place-based factors and health disparities [20, 31, 49, 53, 54]. Place of residence affects one’s perceptions of their surroundings [55, 56] and those perceptions may, in turn, affect the individual’s health [23, 57–61].

Despite sampling methods to ensure that study participants were selected at varying distances from the railyard, minimal heterogeneity among the various respiratory health outcomes was found. This provided a unique setting to determine the relationship between a place-based, psychosocial determinant and respiratory health in a socially- and environmentally- vulnerable population. The minimal heterogeneity in the respiratory effects from the different environmental hazard (residential sampling) regions allowed us to examine how a psychosocial stressor may contribute to a well-supported [30, 62, 63] causal relationship between ambient air pollution exposure and adverse respiratory health.

The lack of heterogeneity of the respiratory health outcome across the three environmental hazard regions, in addition to the smaller sample size within each region, may have contributed to our null findings when determining if differences exist in the strengths of associations between perceived community safety and respiratory illness in our region-specific sensitivity analyses. On the other hand, these findings could also be due to residual confounding. These unaccounted-for factors (e.g. neighborhood green space or social cohesion) may be different within each residential region, as it has been demonstrated that many low-income, minority study populations have diversity in the range of social disadvantages experienced [20, 64]. Although non-significant, there exists a clear trend in the strengthening of the association between perceived community safety and respiratory illness as residential distance to the environmental hazard gets smaller. The individual associations within region A and region B are borderline significant. In Region A, the prevalence of diagnosed respiratory illness was 80% higher among the participants who reported lack of perceived community safety compared to those who did not report a lack of perceived community safety.

Like many other environmental epidemiology studies, our research had some limitations that should be noted. The ENRRICH study was cross-sectional and temporality between psychosocial stressors and respiratory outcomes cannot be determined. More prospective studies in adult, vulnerable populations in this field of research are needed. However, such studies are difficult to conduct due to financial and methodological constraints (tracking bi- and monolingual low-income populations is challenging). Lack of trust in low income neighborhoods may have affected participation; however, given our response rate (87%) we believe that our CBPR approach may have ameliorated this issue to a large degree. Also potentially affecting participation, some residents may not have been available for interviewing during daylight (working) hours or during early evening daylight hours we used for interviewing. Nevertheless, persons who had spent more time in the environment (resulting in higher risk exposure), and thus were more likely to be available to be interviewed, were of particular interest to this study. Another possible limitation is that both respiratory illness and respiratory symptoms were self-reported and thus subject to information bias; however, a compilation of survey items was explored to capture participants who may have underreported due to health care illiteracy or who may not have had a proper diagnosis due to lack of healthcare access [65].

Despite our limitations, our study has notable strengths. Sampled households were randomly selected. Data was collected by trained bi-lingual community members using a community based participatory research approach [40], which contributed to a high response rate, a notable strength, in a majorly Latino low income target population, with many facing immigration challenges. In addition, it is an advantage to be able to assess psychosocial stressors measured at an individual-level rather than solely relying on census block-level data or use of administrative data. Because the impact of a stressor depends on how one experiences, perceives, and/or interprets the event [47, 61], measuring social stressors at a group-level can result in imprecise assessments of psychosocial stress [10].

Furthermore, the consideration of the community member’s concerns as a guide for this analysis, by use of the ENRRICH project’s mixed-methods design, lends itself to two strengths. First, we analyzed an identified salient social stressor in this community across a spatially heterogeneous area, which reduced confounding by other hypothesized, less-salient stressors [10]. Secondly, because the experience and ‘voices’ of community members are crucial for successful place-based interventions [66], this study will be more easily translatable to public health practice in populations that are *socially-, economically- and environmentally*-vulnerable to adverse health outcomes.

Current research and environmental policy has focused too narrowly on air pollutants alone and should be broadened to take into account the cumulative impact of exposures and vulnerabilities encountered by people who live in low-SES, minority neighborhoods [67]. Our study demonstrates that heterogeneity with regards to social vulnerability (i.e. perceptions of community safety) exists within a low-income, minority population and that a non-chemical, yet place-based factor (i.e. perceptions of community safety) may contribute to adverse chronic respiratory health, aside from exposure to a stationary air pollution emission source alone. Future steps for analyses using the ENRRICH study population will be to test interactions between place-based factors and individual perceptions and to determine relevant social buffers for future intervention strategies.

## Conclusions

In conclusion, when living in a low-income community near a goods movement network with high exposure to diesel emission and in a region with routine poor air quality, the added psychosocial stressor of perceiving your residential community as unsafe increases the likelihood of having a doctor diagnosed chronic respiratory illness. Our finding further supports that when trying to elucidate the effect of air pollution on respiratory health, public health professionals and policy makers must take into account a communities’ social, as well as environmental risk, context.

## Additional file

Additional file 1: Project ENRRICH Adult Participant Questionnaire; (survey instrument). (DOCX 58 kb)

## Abbreviations

CBPR: Community-based participatory research
CI: Confidence interval
COPD: Chronic obstructive pulmonary disease
ENRRICH Project: Environmental Railyard Research Impacting Community Health Project
HMO: Health maintenance organization
PR: Prevalence ratio
SBR: San Bernardino Railyard
SES: Socio-economic status
