# Supplementary material for: Are perceptions of community safety associated with respiratory illness among a low-income, minority adult population?
Source: BMC Public Health. 2018 Sep 3;18:1089. doi: 10.1186/s12889-018-5933-4 (PMC6122647; doi:10.1186/s12889-018-5933-4)
Supplement: Supplementary file 1 — Project ENRRICH Adult Participant Questionnaire; (survey instrument). (DOCX 58 kb) [file 12889_2018_5933_MOESM1_ESM.docx]

**IDENTIFICATION NUMBER______________________ Date:_________________**

**Team(Members):___________________________ ZONE___________________**

*The following questions I am going to ask you, will cover a wide range of topics including things such as: gender, employment, health status, diet, exercise, or how you might feel about things in your community, (for example: air quality or safety). The reason for having such a broad range of questions is to give us a better understanding of your health and how your surrounding community ( such as the train yard, traffic, freeways…etc.) may or may not influence your health. The entire process of asking the questions and having you answer usually take about 30 minutes. Do you have any questions before we begin the survey? Then let’s begin!*

**The first few questions I am going to ask you will be about basic demographics including, gender, race, marital status….etc.**

**DEMOGRAPHICS**

1. What is your gender?

[ ]_1_ Female

[ ]_2_  Male

2. What race/ethnicity do you identify with?

[ ]_1_ White

[ ]_2_ African-American, Black

[ ]_3_ Hispanic or Latino

[ ]_4_ Asian

[ ]_5_ Other race:

*(please specify)*

3. What is the primary language spoken in your home?

[ ]_1_ English

[ ]_2_ Spanish

[ ]_3_ Other_____________________________________

*(please specify)*

4. What is your year of birth?

YYYY

5. What type of health insurance do you currently have?

[ ]_1_ I don’t have any health insurance

[ ]_1a_ How long ago has it been since you last had health insurance?________

[ ]_2_  Private Insurance, Blue Cross, HMO

[ ]_3_ Medicare/Medicaid/Medical

[ ]_4_  Champus/ Champus VA/other military

[ ]_5_  Other type of insurance:

*(please specify)*

6. What is your highest level of education?

[ ]_1_ Grade School or Less Education

[ ]_2_ High school diploma or equivalent (trade school certificate)

[ ]_3_ Some college or Vocational, Business or Trade School

[ ]_4_  Associate or Bachelors college degree

[ ]_5_ Masters or Doctoral degree

7. What is your marital status?

[ ] _1_ Single, never married [ ] _5_  Common law marriage/Live together

[ ] _2_ Married

[ ] _3_ Widowed

[ ] _4_ Divorced/Separated

**HOUSE CHARACTERISTICS**

Now I am going to ask you questions about where you have lived including your where you live now and where you have lived previously. We need to know where you have lived to know what types of different air pollutants you may have been breathing into your lungs and it varies across the city, state or even the US.

8. How long have you lived at your current address?

[ ]_1_ All your life (skip to question #11)

[ ]_2_ Less than 1 year

[ ]_3_  1-5 years

[ ]_4_  5-10 years

[ ]_5_ 11+ years

9. What was the address of the city you lived in **before the current address**?

Address:____________________________________

City: ____________________________________

State:

Zipcode:___ ___ ___ ___ __

Country__________________________________

10) How long did you live at the previous address?____________________________

*Please specify*

11. How many people live in your current house, including yourself?

[ ]_1_ I live alone

[ ]_2_  2 people

[ ]_3_ 3 people to 5 people

[ ]_4_ 6 or more people

12. How many people in your house are under the age of 13 years?

[ ]_1_ No one under the age of 13 years

[ ]_2_ 1 child under 13 years

[ ]_3_ 2 children under 13 years

[ ]_4_  3 children under 13 years

[ ]_5_ 4 or more children under 13 years

13. What type of **heating** do you use inside your house? (check all that apply)

[ ]_1_  I don’t have any type of heating

[ ]_2_  Only a fireplace

[ ]_3_ Natural gas heater

[ ]_4_  Other type of heating:

*Please specify*

14. What type of **air cooling** do you use inside your house? (check all that apply)

[ ]_1_ Open the windows

[ ]_2_ Air conditioner window unit

[ [_3_  Central air conditioning (refrigerating type)

[ [_4_  Portable fan or ceiling fan

[ ]_5_ Other type:

*Please specify*

15. What type of cooking stove do you use inside your house?

_______________________________

*Please specify*

16. On average how many hours in a week do you typically spend outdoors in your community (not including the times you are exercising or working hard)?

_____________________hrs. per week

*Please specify*

17. On average how many hours in a week do you exercise vigorously or do heavy physical labor (e.g. jogging, heavy lifting…etc.) outdoors in your community?

_____________________hrs. per week

*Please specify*

18. During the last 7 days, have you exercised or done any moderate physical activities either indoors or outdoors (moderate physical activities make you breathe harder such as bicycling, dancing, swimming..etc.)?

[ ]_1_ No, (go to question # 22)

[ ]_2_  Yes

19. How many days during the week did you exercise or do these moderate activities?

________________days per week

20. On one of the days that you exercised or did moderate activities, how much time would you estimate that you spent doing this activity?

_____________________hours per day ___________________________minutes per day

21. Have you exercised or done moderate activities within the last 2 hours?

[ ]_1_ No

[ ]_2_ Yes

**Now I am going to ask you a few questions about any smoking you might or might not do. And if there are people around you that might smoke as well:**

**PERSONAL SMOKING HISTORY**

22. Have you ever regularly smoked cigarettes, cigars or a pipe?

[ ]_1_ No (*Please skip to question # 30)*

[ ]_2_ Yes, if yes then what do you smoke? (check all that apply)

[ ]_a_ Cigarettes

[ ]_b_ Cigars

[ ]_c_ Pipe

23. At what age did you first start smoking regularly? Years

24. Are you currently smoking?

[ ]_1_ No

[ ]_2_ Yes *(please skip to question # 26)*

25. At what age did you stop smoking? Years

26. Approximately how many years, in total, have you regularly smoked (not counting the times when you had quit)? ______________ Years

27. During most of the time that you regularly smoked cigarettes, how many cigarettes did you usually smoke each day?

[ ]_1_ Less than 5 cigarettes per day

[ ]_2_  5-14 (1/2 pack) per day

[ ]_3_ 15-44 (1-2 packs) per day

[ ]_4_  45+ (2 ½ packs or more) per day

28. During most of the time that you regularly smoked cigars, how many cigars did you usually smoke?

[ ]_1_ About 1 cigar per day

[ ]_2_  About 1 cigar per week

[ ]_3_ About 1 cigar per month

29. During most of the time that you regularly smoked a pipe, how often did you usually smoke?

[ ]_1_ More than once a day

[ ]_2_ About once a day

[ ]_3_  About once a week

[ ]_4_ About once a month

**PASSIVE (ENVIRONMENTAL) EXPOSURE TO TOBACCO SMOKE**

30. As an adult (18 years of age or older), have you ever **lived** for 6 months or more with someone who smoked inside your house?

[ ]_1_ No *(please skip to question # 32)*

[ ]_2_  Yes, in the past

[ ]_3_ Yes, currently

31. How many years did you live with someone who smoked in the house, if less than one year indicate how many months?

_____________months or ______________years

32. As an adult (18 years of age or older), have you ever **worked** for 6 months or more with someone who smoked inside your workplace?

[ ]_1_ No *(please skip to question # 34)*

[ ]_2_ Yes, in the past

[ ]_3_ Yes, currently

33. How many years did you work with someone who smoked in the workplace, if less than one year indicate how many months?

_____________months or ______________years

**Next I am going to ask you a few questions about your employment or your previous employment over the years:**

**EMPLOYMENT**

34. What is your current employment status?

[ ]_1_ Unemployed (skip to question # 36 )

[ ]_2_ Employed part time

[ ]_3_ Employed full time

[ ]_4_ Retired and not working

[ ]_5_ Retired but working part time or full

[ ]_6_ Student

35. On an average day how much time do you spend driving or commuting to and from work or school?

[ ]_1_ I do not commute

[ ]_2_ 0 to less than 1 hour

[ ]_3_ 1 to less than 2 hours

[ ]_4_ 2 hours or more

36. What is your usual or main occupation (Do not write “retired” – if retired or not now working, give your usual occupation when you were working)

Job title:

Type of business or industry:

37. How many years have you been employed in this occupation? Years

38. What would you estimate is your average annual household (all family members combined) income?

[ ]_1_ Less than $10,000 per year

[ ]_2_ $11,000 - $19,000 per year

[ ]_3_ $20,000 - $29,000 per year

[ ]_4_ $30,000 - $49,000 per year

[ ]_5_ $50,000 - $74,000 per year

[ ]_6_  More than $74,000 per year

39. Do you work or have you ever worked for any railyard (BNSF, Union Pacific..etc.)?

[ ]_1_ No (if no, skip to question #44)

[ ]_2_ Yes, what was or is the field you worked in? (check all that apply)

[ ]_a_ Locomotive engineer

[ ]_b_ Railroad conductor

[ ]_c_ Railroad brake operators

[ ]_d_ Yard master/worker

[ ]_e_ Switch operators

[ ]_f_ Other___________________________

40. How long have you worked for the railyard?____________months or __________years

41. What was the name of the railyard, including the city?_______________________

42. Do you currently work for the BNSF San Bernardino railyard?

[ ]_1_ No (if no, skip to question # 44)

[ ]_2_ Yes

43. How long ago did you work for the railyard?_________months or ____________years

44. Have you ever worked as a driver for the trucking industry?

[ ]_1_ No (if no, skip to question #48)

[ ]_2_ Yes

45. How long have you worked as a driver in the trucking industry?________ months or ____________years

46. Do you currently work as a driver for the trucking industry?

[ ]_1_ No

[ ]_2_ Yes (if yes, skip to question # 48)

47. How long ago did you work as a driver for the trucking industry?________ months or ____________years

**********************************************************************************

**Now I would like to ask you some questions about your health and use of health care services:**

**COUGH**

48. When you wake up for the day, do you find that you frequently have to cough first thing in the morning?

[ ]_1_ No

[ ]_2_  Yes

49. Do you find that you frequently cough at other times during the day or night?

[ ]_1_ No (if answered no to question #48 and #49 then go to question # 52)

[ ]_2_ Yes

50. Thinking over this past year, do you feel that you coughed on most days for at least 3 months or more of the year?

[ ]_1_ No

[ ]_2_ Yes

51. For how many years have you had a cough?

[ ]_1_ Never

[ ]_2_ Less than 1 year

[ ]_3_ More than 1 but less than 2 years

[ ]_4_ 2-5 years

[ ]_5_ More than 5 years

**SPUTUM**

52. When you wake up for the day do you frequently bring up phlegm, sputum, or mucus from your chest first thing in the morning?

[ ]_1_ No

[ ]_2_ Yes

53. Do you frequently bring up phlegm, sputum, or mucus from your chest at other times during the day or night?

[ ]_1_ No (if no to question #52 and #53 then skip to question #56)

[ ]_2_  Yes

54. Thinking over this past year, do you feel that you bring up phlegm, sputum, or mucus from your chest on most days for 3 months or more of the year?

[ ]_1_  No

[ ]_2_ Yes

55. For how many years do you think you have you raised phlegm, sputum, or mucus from your chest?

[ ]_1_ Never

[ ]_2_  Less than 1 year

[ ]_3_ More than 1 but less than 2 years

[ ]_4_ 2-5 years

[ ]_5_ More than 5 years

**WHEEZING**

56. Does your breathing ever sound wheezy or whistling?

[ ]_1_ No

[ ]_2_ Yes

57. Does your chest ever sound wheezy or whistling when you have a cold?

[ ]_1_ No

[ ]_2_ Yes

58. Does your chest ever sound wheezy or whistling when you don’t have a cold?

[ ]_1_ No (skip to question #60)

[ ]_2_  Yes

59. Does your chest ever sound wheezy or whistling for the majority of the day or night?

[ ]_1_ No

[ ]_2_ Yes

60. Have you ever had attacks of shortness of breath with wheezing?

[ ]_1_ No *(please skip to question #63)*

[ ]_2_ Yes

61. Have you had 2 or more such episodes?

[ ]_1_ No

[ ]_2_ Yes

62. Have you ever required medicine or treatment for the(se) attack(s)?

[ ]_1_ No

[ ]_2_ Yes

**BREATHLESSNESS**

63. Are you troubled by shortness of breath when hurrying on level ground or walking up a slight hill?

[ ]_1_ No

[ ]_2_ Yes

64. Do you get short of breath when walking at a normal pace with other people of your own age on for example a level sidewalk?

[ ]_1_ No

[ ]_2_ Yes

**RESPIRATORY ILLNESS**

65. During the past 12 months, how often were you unable to do your usual activities because of illnesses (for example: chest colds, bronchitis, or pneumonia)

[ ]_1_ None

[ ]_2_ 1 time

[ ]_3_ 2-5 times

[ ]_4_ More than 5 times

66. Has a doctor ever told you that you had asthma, some kind of bronchial condition, or emphysema?

[ ]_1_ No

[ ]_2_ If yes, please check which conditions:

[ ]_a_ Asthma

[ ]_b_ Bronchial condition

[ ]_c_ Emphysema

**ADDITIONAL HEALTH**

67. Do you frequently suffer from headaches?

[ ]_1_ No

[ ]_2_ Yes, if yes please check how often

[ ]_a_ Every day or every few days

[ ]_b_ Once a week

[ ]_c_ Once a month

[ ]_d_ Other__________________

*Please specify*

68. Do you frequently suffer from bloody noses?

[ ]_1_ No

[ ]_2_ Yes, if yes please check how often

[ ]_a_ Every day or every few days

[ ]_b_ Once a week

[ ]_c_ Once a month

[ ]_d_ Other__________________

*Please specify*

69. Do you frequently have itchy or watery eyes?

[ ]_1_ No

[ ]_2_ Yes, if yes please check how often

[ ]_a_ Every day or every few days

[ ]_b_ Once a week

[ ]_c_ Once a month

[ ]_d_ Other__________________

*Please specify*

70. Have you ever experienced ringing in either one or both of your ears, while you were in a quiet area?

[ ]_1_ No

[ ]_2_ Yes

71. Have you ever experienced other noises in either one or both of your ears (i.e. clicking, hissing, roaring or buzzing while you were in a quiet area?

[ ]_1_ No, (*please skip to question #73 if you answered no to 70 and 71)*

[ ]_2_ Yes

72. Is the noise (i.e. ringing, clicking, buzzing…etc.) in your ears fairly constant?

[ ]_1_ No

[ ]_2_ Yes

73. Has a physician or any other medical professional told you that you have hearing loss?

[ ]_1_ No

[ ]_2_ Yes

The next question I need to ask you is about pregnancy. I need to find out whether or not you are pregnant or recently had a baby, because it is important to understand how many infants may live in your community as their lungs are very sensitive to their surrounding environment.

74. What is your current pregnancy status? (check all that apply)

[ ]_1_ Pregnant

[ ]_2_ Recently had a baby less than 1 year ago

[ ]_3_ None of the above

The next two questions I am going to ask you are about your height and weight. By knowing your height and weight we are better able to understand your overall health and that of your lungs.

75. What do you think is your approximate weight?

____________________lbs

76. What do you think is your approximate height?

______ ft. _______________in.

**HEALTH CARE UTILIZATION**

77. Is there a place that you usually go when you are sick or need advice about your health?

[ ]_1_ No

[ ]_2_ Yes, (please check all the locations you go to)

[ ]_a_ Doctor’s office/Clinic

[ ]_b_ County Public Health Department Clinic

[ ]_c_ Emergency Room

[ ]_d_ Other Place (please specify)_________________________________

78. How long do you think it has been since you last saw a doctor about your own health?

[ ]_1_ One year ago or less

[ ]_2_ More than 1 year but less than 2 years

[ ]_3_ More than 2 years but less than 5 years

[ ]_4_ More than 5 years ago

[ ]_5_ Never

79. How many times over the past 12 months have you visited a health care provider (such as your doctor) for a respiratory or heart condition?

[ ]_1_ Never

[ ]_2_ 1-2 times over past 12 months

[ ]_3_ 3-4 times over past 12 months

[ ]_4_ 5 or more times over past 12 months

80. How many times over the past 12 months have you been seen in a hospital emergency department for a respiratory or heart condition?

[ ]_1_ Never

[ ]_2_ 1-2 times over past 12 months

[ ]_3_ 3-4 times over past 12 months

[ ]_4_  5 or more times over past 12 months

81. How many times over the past 12 months have you been hospitalized for a respiratory or heart condition?

[ ]_1_ Never

[ ]_2_  1-2 times over past 12 months

[ ]_3_  3-4 times over past 12 months

[ ]_4_ 5 or more times over past 12 months

82. During the past 12 months how many days of work did you miss due to a respiratory or heart condition? (if unemployed, enter a zero)

_____________________days.

83. Do you use a physician-prescribed inhaler?

[ ]_1_ No *(please skip to question # 86)*

[ ]_2_ Yes________________________________

Please list name of inhaler medication

84. How many years (or months, if less than one year) have you used such an inhaler?

Months Years

85. How many times per week do you feel you have to use an inhaler because of difficulty breathing?

[ ]_1_ None

[ ]_2_ Occasional use, less than 1 time per week on average

[ ]_3_ 1-2 times per week

[ ]_4_ 3-5 times per week

[ ]_5_ 1 time per day

[ ]_6_ 2 or more times per day

86. How often does your health status hinder you from enjoying your life?

[ ]_1_ None at all

[ ]_2_ Only slightly

[ ]_3_ Monthly

[ ]_4_ Weekly

[ ]_5_ Daily

87. How would you describe your general health?

[ ]_1_ Excellent

[ ]_2_ Good

[ ]_3_ Fair

[ ]_4_  Poor

88. Has a physician ever told you that you have any of the following conditions? (check all that apply)

[ ] _1_ Diabetes [ ] _9_ High blood pressure

[ ] _2_ Kidney Disease [ ] _10_ Chronic Obstructive Pulmonary Disease (COPD)

[ ] _3_ Angina [ ] _11_ Migraines

[ ] _4_ Anemia [ ] _12_ High cholesterol

[ ] _5_ Cancer_____________________

*Please specify what type of cancer (example: lung cancer, or bladder cancer…etc.)*

[ ] _6_ Stroke

[ ] _7_ Allergies [ ] _13_ GERD/acid reflux

[ ] _8_ Cystic fibrosis [ ] _14_ Sinusitis

[ ] _15_ Other___________________________

*Please specify*

[ ] _16_ None of the Above

89. Within the past 12 months were there medical services you needed but could not get?

[ ]_1_ No

[ ]_2_ Yes , if yes, please indicate the reasons for not being able to get:

[ ]_a_ No transportation

[ ]_b_ Lack of money

[ ]_c_ Lack of insurance

[ ]_d_ Not able to take time off from work

[ ]_e_ No child care

[ ]_f_ Other_______________________________

*Please specify*

90. Within the past 12 months were there prescription medications you needed but could not get?

[ ]_1_ No

[ ]_2_ Yes , if yes, please indicate the reasons for not being able to get:

[ ]_a_ Lack of money

[ ]_b_ Lack of insurance

[ ]_c_ Other_______________________________

*Please specify*

***************************************************************************

**DIET**

**Now I am going to ask your questions about your usual diet:**

91. On average, how many times per week would you estimate that you eat fruit (any kind, fresh, canned, dried..etc.)?

________________________times per week or

_________________________times per month

92. On average how many times per week would you estimate that you eat vegetables like green salad, green beans, potatoes..etc. (do not include fried potatoes).

______________________times per week or

_______________________times per month

93. On average how many times per week would you estimate that you drink regular soda or pop or juice?

______________________times per week or

_______________________times per month

94. On average how many times per week would you estimate that you eat sweets like cookies, cake, pies, brownies, ice cream or other frozen desserts, candy …etc.?

______________________times per week or

_______________________times per month

95. On average how many times per week would you estimate that you eat or get take-out food from a fast food restaurant (McDonalds, Del Taco, Burger King, El Pollo Loco, KFC…etc.)?

_________________________times per week or

_______________________times per month

Now I am going to read to you two statements that people have made about their food situation. For each please tell me whether the statement describes something that was often true, sometimes true or never true for you and your household over the past 12 months:

96. “The food that I/we bought just didn’t last and I/we didn’t have money to get more.”

[ ]_1_ Often true

[ ]_2_ Sometimes true

[ ]_3_ Never true

97. “I or my family couldn’t afford to eat balanced meals.”

[ ]_1_ Often true

[ ]_2_ Sometimes true

[ ]_3_ Never true

**Now I am going to ask you a few questions about drinking alcohol, wine, beer or hard liquor :**

98. Do you currently drink alcoholic beverages (beer, wine, hard liquor), even if only occasionally?

[ ]_1_ No *(please skip to question # 103)*

[ ]_2_ Yes , (please check all that apply)

[ ]_a_ Beer

[ ]_b_ Wine

[ ]_c_ Hard liquor

99. Total time that you have drank alcoholic beverages:

[ ]_1_ Less than 1 year

[ ]_2_ 1-5 years

[ ]_3_ 6-9 years

[ ]_4_  10-19 years

[ ]_5_ 20 or more years

100. On average when you drank **beer** how much would you estimate that you drank (12 oz, or 1 can of beer) ?

[ ]_1_  Less than 3 drinks per month

[ ]_2_ 1-2 drinks per week

[ ]_3_ 3-6 drinks per week

[ ]_4_ 1 or more drinks per day

101. On average when you drank **wine** how much would you estimate that you drank (6 oz, about 1 glass)?

[ ]_1_  Less than 3 drinks per month

[ ]_2_ 1-2 drinks per week

[ ]_3_ 3-6 drinks per week

[ ]_4_ 1 or more drinks per day

102. On average when you drank hard liquor (like Tequila, Jack Daniels..etc.) how much would you estimate that you drank (1 oz about 1 shot)?

[ ]_1_  Less than 3 drinks per month

[ ]_2_ 1-2 drinks per week

[ ]_3_ 3-6 drinks per week

[ ]_4_ 1 or more drinks per day

103. Have you had anything to drink or eat (except water) within the last two hours?

[ ]_1_ No

[ ]_2_ Yes: _____________________________________________

*Please specify*

*********************************************************************************

**Now I would like to ask you a couple of questions about the noise level in your community:**

**COMMUNITY NOISE**

104. What do you think is the level of noise outside of your home, which you hear from inside your home while the windows and doors are closed (not noise coming from kids or people hanging out together..etc.)?

[ ]_1_ Quiet

[ ]_2_ Mostly quiet/Slight noise

[ ]_3_ Moderate noise

[ ]_4_ Extreme noise

105. Where do you think the majority of the noise in your community is coming from? (check all that apply)

[ ]_1_ Trucks

[ ]_2_ Railyard

[ ]_3_ Businesses

[ ]_4_ General traffic

[ ]_5_ Other___________________________________________________

*Please specify*

106. What time of day is the noise the loudest in your community? (check all that apply)

[ ] _1_ Morning [ ] _3_ Afternoon

[ ] _2_ Evening [ ] _4_ Night time

[ ] _5_ All times of day and night

**********************************************************************************

**Now I am going to read to you statements people have made about their community or health. For each phrase I would like for you to tell me whether the statement describes something that you strongly agree with, agree with, neither agree or disagree but neutral, disagree or strongly disagree:**

1=Strongly Agree

2=Agree

3=Neutral, Neither disagree nor agree

4=Disagree

5=Strongly Disagree

**Safety**

107._____I feel safe walking in my community, day or night.

108._____Violence or crime is not a problem in my community.

**Social Cohesion**

109._____People in my community generally get along with each other.

110._____People in my community can be trusted.

111._____I often see children playing outside in the community.

**Walking environment**

112._____Local facilities in my community offer many opportunities to get exercise.

113._____The trees in my community provide enough shade.

114._____My community has heavy traffic.

115._____I often see other people walking in my community.

116._____There is a lot of trash and litter on the street in my community.

117._____I believe it doesn’t matter what time of day I exercise outside.

118._____The air in my community does not pose a risk to my health or that of my family.

**Availability of healthy foods**

119._____A large selection of fresh fruits and vegetables is available in my community.

120._____The fresh fruits and vegetables available in my community are of high quality.

121._____There are many opportunities to purchase fast foods in my community.

**Perception**

122._____I can do just about anything I really set my mind to.

123._____I often feel helpless in dealing with the problems of life.

124._____I am concerned about the air quality in my neighborhood.

**Sleep**

125._____I tend to wake up in the middle of the night or am woken up when I should be

sleeping.

126._____I have trouble sleeping because I cough or cannot breathe comfortably.
127._____I tend to use medication to help me fall asleep.

128._____I tend to sleep 8 or more hours per night.

129.____The noise from my community keeps me awake or wakes me up in the middle of the

night.

**COMMUNITY CHANGES**

**We are coming close to the end of our questioning and I have a few more really important questions that I would like to ask you. The following questions are about making your community an even safer and better place:**

130. What changes would you like to see happen to improve within your community?

_______________________________________________________________

131. What changes would you like to see in your community specific to improving the air quality within your community?

_______________________________________________________________

132. What changes do you feel the government or city officials should make for your community to improve air quality?

____________________________________________

133. What changes do you feel the San Bernardino Railyard should make for your community to improve air quality?

_______________________

134. What changes do you feel that you could make to improve the air quality in your community?

________________________________

*****************************************************************************

**We are at the end of our survey questions:**

Thank you, I really appreciated your time and cooperation. You have helped with a very important survey. Are there any concerns or questions that you would like to discuss with us? (please document in the space below) If you have additional questions later on, you can contact people listed on your informed consent document that we have left with you. Thank you and good bye.
